# Easy Identification of Optimal Coronal Slice on Brain Magnetic Resonance Imaging to Measure Hippocampal Area in Alzheimer's Disease Patients

**DOI:** 10.1155/2020/5894021

**Published:** 2020-09-23

**Authors:** P. Zach, A. Bartoš, A. Lagutina, Z. Wurst, P. Gallina, T. Rai, K. Kieslich, J. Riedlová, I. Ibrahim, J. Tintěra, J. Mrzílková

**Affiliations:** ^1^Department of Anatomy, Third Faculty of Medicine, Charles University, Prague, Czech Republic; ^2^National Institute of Mental Health, Topolová 748, 250 67 Klecany, Czech Republic; ^3^Department of Neurology, Third Faculty of Medicine, University Hospital Kralovske Vinohrady, Charles University, Prague, Czech Republic; ^4^Department of Surgery and Translational Medicine, Neurosurgery Unit, Florence School of Neurosurgery, University of Florence, Florence, Italy; ^5^Institute of clinical Clinical and Experimental Medicine, Prague, Czech Republic

## Abstract

**Introduction:**

Measurement of an- hippocampal area or volume is useful in clinical practice as a supportive aid for diagnosis of Alzheimer's disease. Since it is time-consuming and not simple, it is not being used very often. We present a simplified protocol for hippocampal atrophy evaluation based on a single optimal slice in Alzheimer's disease.

**Methods:**

We defined a single optimal slice for hippocampal measurement on brain magnetic resonance imaging (MRI) at the plane where the amygdala disappears and only the hippocampus is present. We compared an absolute area and volume of the hippocampus on this optimal slice between 40 patients with Alzheimer disease and 40 age-, education- and gender-mateched elderly controls. Furthermore, we compared these results with those relative to the size of the brain or the skull: the area of the optimal slice normalized to the area of the brain at anterior commissure and the volume of the hippocampus normalized to the total intracranial volume.

**Results:**

Hippocampal areas on the single optimal slice and hippocampal volumes on the left and right in the control group were significantly higher than those in the AD group. Normalized hippocampal areas and volumes on the left and right in the control group were significantly higher compared to the AD group. Absolute hippocampal areas and volumes did not significantly differ from corresponding normalized hippocampal areas as well as normalized hippocampal volumes using comparisons of areas under the receiver operating characteristic curves.

**Conclusion:**

The hippocampal area on the well-defined optimal slice of brain MRI can reliably substitute a complicated measurement of the hippocampal volume. Surprisingly, brain or skull normalization of these variables does not add any incremental differentiation between Alzheimer disease patients and controls or give better results.

## 1. Introduction

Rating of medial temporal lobe atrophy is recommended in all current diagnostic guidelines [1]. However, it is seldom used in routine clinical dementia assessment. Total brain volume and volume-to-brain ratio in comparison to hippocampal and temporal horn measurements in traumatic brain injury showed hippocampal atrophy and temporal horn enlargement. The hippocampus and temporal horn volumes were inversely correlated in the group with traumatic brain injury [2]. The inverse relationship between the hippocampus and adjacent ventricle or the third ventricle is also used for detection of Alzheimer's disease [3–9]. The measure of temporal atrophy (radial width of the temporal horn (rWTH)) was used to distinguish its asymmetry in AD patients [10]. Anatomical mapping of structural changes in AD showed more sensitive temporal horn expansion compared to hippocampal atrophy, but both maps correlated with clinical findings [11].

Our goal was to define single the most appropriate slice to measure the absolute hippocampal area, compare it with the hippocampal volume measurement, and find out whether normalization of the hippocampus to the area of the brain section, total intracranial volume, or skull area is useful in differentiation of AD patients and controls on MRI.

## 2. Material and Methods

### 2.1. Participants

Brain magnetic resonance imaging (MRI) and the Mini-Mental State Examination (MMSE) were examined in 80 individuals during our validation and normative study of the MMSE [12]. We included two groups of participants. The first group of patients (*n* = 40) was diagnosed with dementia due to Alzheimer's disease (AD) according to the National Institute on Aging-Alzheimer's Association (NIA-AA) criteria [13] at memory clinic of AD center, Department of Neurology, Charles University, Prague, Czech Republic. The second group of normal elderly controls (NC) (*n* = 40) had normal MMSE scores using our Czech norms and cut-offs for mild AD [12]. They were recruited mainly at Universities of Third Age (educational courses for seniors) or were spouses of the patients. Sociodemografic characteristics and cognitive scores of both groups are compared in (Table 1).

All participants signed informed consent. The research was approved by the Ethics Committee of the Prague Psychiatric Center/National Institute of Mental Health.

### 2.2. Acquisition of Magnetic Resonance Imaging

Brain MRI were acquired in 3D with scanner model SIEMENS TrioTim and software Syngo MR B13 4VB13A. Magnetic field strength was 3 T, voxel size 0.85∗0.85∗0.85 mm, slice thickness 0.85 mm, repetition time 2000 ms, echo time 4.73 s, scanning sequence GR/IR, acquisition matrix 320∗384, and flip angle 10°. Participants were imaged at the Institute of Clinical and Experimental Medicine (IKEM), Prague, Czech Republic.

### 2.3. Specification of Optimal Brain Slice for Two-Dimensional Hippocampal Area Measurement Using Manual Delineation

**Figure 1:**
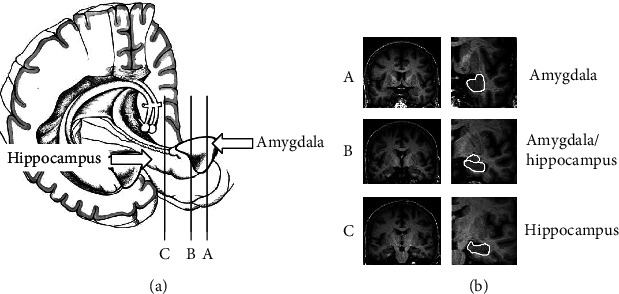
Three sections through the hippocampus and amygdala show their different proportions. Slice A through amygdala is the first notice/signal during viewing MRI slices from anterior/front to back. Slice B contains both hippocampal and amygdala areas on the slice, and it is not appropriate yet for evaluation. The optimal slice for hippocampal area measurement is slice C with the hippocampus only, without any part of amygdala. It is the first slice going back where amygdala disappears.

**Figure 2:**
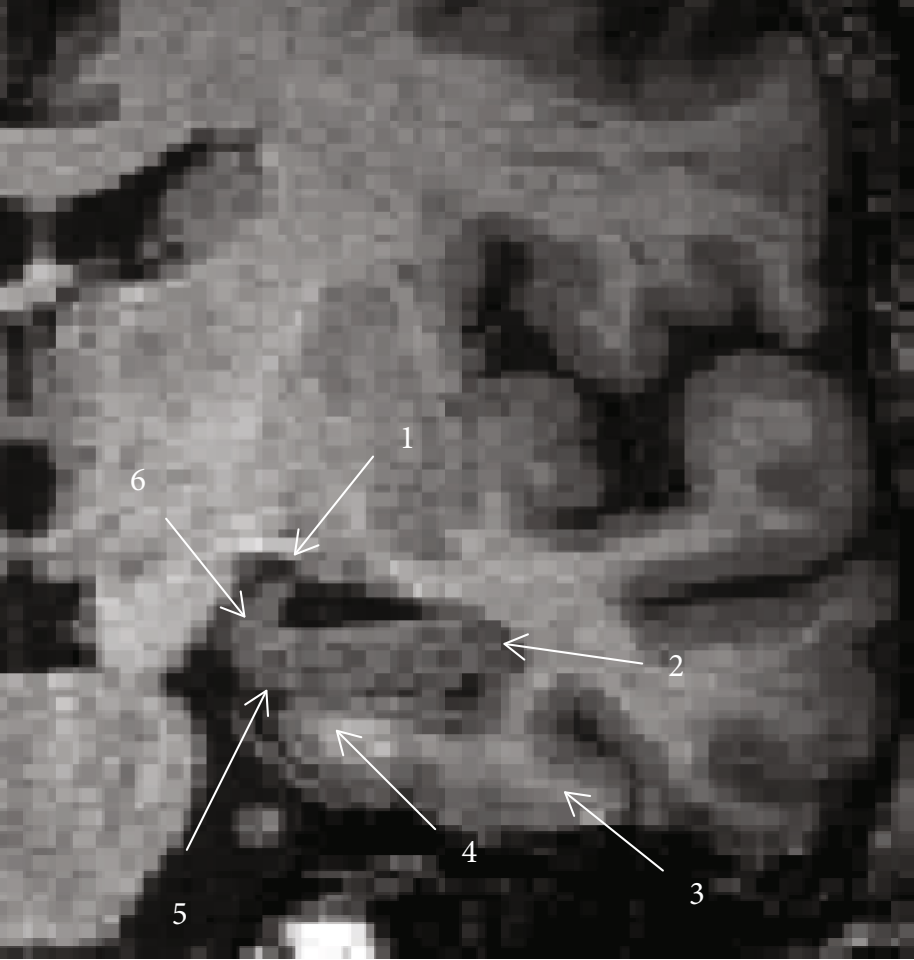
A detailed view of left mediotemporal structures on the optimal slice of brain MRI. Anatomical structures are labeled as follows: 1: hippocampal fimbria; 2: alveus; 3: parahippocampal gyrus; 4: subiculum; 5: hippocampal fissure; 6: uncus of the parahippocampal gyrus.

In order to define hippocampal shrinkage on MRI for the clinical applications (dementia severity and its progression), we designed a protocol from a single coronal brain slice. We found gray matter located inside the temporal lobe (at the caudal part of the temporal horn of the lateral ventricle) by viewing the coronal slice on MRI in ventrodorsal orientation (Figure 1). Then, we located the amygdalar complex in the temporal lobe, below the lateral horn of the lateral ventricle. When we looked through coronal slices located more dorsally, we could see the hippocampus positioned below the *amygdalar complex*. With the increase in coronal slice numbers, we could see *alveus* in the caudal and lateral part of the *hippocampus. Fimbria hippocampi* was located on the top surrounded by several *amygdalar nuclei*. We considered the optimal coronal slice where *amygdalar nuclei* were no longer visible (at the level of *fimbria hippocampi*) so that we could observe the full extent of the *hippocampus*: *alveus*, *dentate gyrus*, and *fimbria fornicis*, located on the *parahippocampal gyrus* (Figure 2). In case of hippocampal atrophy, there was a significant reduction of both the gray and the white matter so that *fissura hippocampi* became clearly visible horizontally at the transition between the *hippocampus* and *subiculum* (part of *parahippocampal gyrus*).

### 2.4. Three-Dimensional Volumetry Using FreeSurfer Analysis

Images were processed using the most recent version of FreeSurfer (v6.0) software http://surfer.nmr.mgh.harvard.edu which creates virtual 3D reconstruction of human brain stacking slices of MR images in 3D space [14]. DICOM MR images from the MR scanner of the controls and the AD patients were converted into FS program ∗.mgz files by mri_convert command. Full-image reconstruction (cortical and subcortical areas, brain stem, cerebrospinal fluid, ventricles, and white matter) was done by recon-all command. Then, brain structures were segmented due to different contrasts of brain tissue mapped on the Talairach atlas template derived from numerous human brains by creators of the software. The next step was the calculation of volumetric values of different brain structures. Volume datasets for statistical analysis were taken from /stats directory (aseg.stats, lh aparc.stats, etc.) within the FS program and exported into Statistica v.10 software. Two volumetric values were measured and calculated—an absolute volume of the hippocampus on the left and right and total intracranial volume (TIV).

### 2.5. Normalization of Hippocampal Area Measurements

The absolute area of the hippocampus in cm^2^ separately for the left and right sides from a single optimal slice and absolute area of the brain and skull slice in the coronal section at the level of commissura anterior (CA) were manually delineated and calculated by an experienced neuroanatomist (JM) using FiJi (ImageJ software suite, https://imagej.net/Fiji).

The hippocampal area was normalized to the coronal section of the whole brain (and also the skull, unpublished data) at the level of the CA (in cm^2^), separately for the left and right hippocampus, as the ratio of the hippocampal area in the optimal slice to the brain area at CA, multiplied by 100.

### 2.6. Normalization of Hippocampal Volume Measurements

The absolute volume of the hippocampus as well as total intracranial volume (TIV) in cm^3^ was obtained by automated brain segmentation using FS (version 6.0 for Linux) [14, 15].

Intracranial volume, sometimes referred to as TIV, calculated from FreeSurfer (FS) was used for brain normalization, as described, e.g., in [16, 17]. We normalized the absolute hippocampal volumes to TIV separately for the left and right hippocampus as the ratio of hippocampal volume to TIV, multiplied by 100.

### 2.7. Statistics

A *T*-test for independent groups was used to calculate differences between the control and AD groups (grouping factor) and left and right sides (variables). The *T*-test was calculated separately for absolute hippocampal areas and volumes, hippocampal areas normalized to brain areas at CA and hippocampal volumes normalized to TIV. The *T*-test was used also for calculation of differences in demographic characteristics between the control and AD groups (mean ± standard deviation). Cohen's *d* test, AUC-ROC, and comparison of ROC curves evaluated differences between absolute and normalized measurements, separately for the left and right sides. The *T*-test was calculated in Statistica v.10 software, Cohen's *d* test in online Effect Size Calculator (https://lbecker.uccs.edu/), and AUC-ROC test in MedCalc v. 19.2.1. Statistical significance was accepted at *p* ≤ 0.05.

## 3. Results

Table 1 shows that the AD patients were matched with the controls regarding age, education and sex, and significantly differed in MMSE scores.

### 3.1. Absolute Hippocampal Area and Volume

Hippocampal areas in the optimal slice on the left and right in the control group were significantly higher than those in the AD group (*p* < 0.001). Hippocampal volumes on the left and right in the control group were significantly higher than those in the AD group (*p* < 0.001) (Table 2).

### 3.2. Normalized Hippocampal Area and Volume

Normalized hippocampal areas on the left and right in the control group were significantly higher compared to the AD group (*p* < 0.001). Normalized hippocampal volumes on the left and right in the control group were significantly higher compared to the AD group (*p* < 0.001) (Table 2).

### 3.3. Comparison of Absolute and Normalized Hippocampal Areas and Volumes

Normalized hippocampal areas as well as normalized hippocampal volumes did not differ significantly from corresponding absolute hippocampal areas and volumes (*p* > 0.5) (Table 3).

## 4. Discussion

Our results show that hippocampal shrinkage in the AD patients could be reliably evaluated from the single coronal slice of the brain on the MRI and without normalization to the TIV or other brain measures. We combined manual and automated (FS) delineation of the hippocampus. It was found that there is high reliability and agreement between FreeSurfer and manual hippocampal protocols [18]. Our slice is at the level of the memory processing (ventral hippocampus) [19] but not at the level of 3D spatial navigation (dorsal hippocampus) [20]. Other studies used several slices and stages of Alzheimer's disease development but without having MRI slices precisely defined by space position of the anatomical structures [21]. These slices often seem to be localised at the level of the dorsal hippocampus.

Numerous protocols in clinical studies with Alzheimer's disease patients often use the hippocampus to brain TIV normalization [22, 23]. However, our results show that normalization is not necessary in order to evaluate hippocampal shrinkage as part of diagnosis. Possible mistake of total intracranial volume estimation by FS and suggestion for evaluation of intracranial volume by two intracranial areas and width are mentioned in [24, 25]. No effects of age-related changes on the MRI were observed in the temporal lobe width and temporal horn width measurements both on right and left sides [26]. Temporal horn volumes and temporal horn indexes measurements in AD were significant in AD compared to controls but not in MCI [4]. On the other hand, temporal horn expansion was found more reliable to predict conversion from MCI to AD than hippocampal volume alone because of smaller changes in it compared to the size of the temporal horn [27]. Other variants of a 2D single brain slice from which we could derive measurements of *hippocampus* atrophy represent study that measured manually 3 regions on coronal MRI at the level of the interpeduncular fossa to calculate the Medial Temporal Atrophy index [7]. Similarly, our design offers only one simple and easily distinguishable marker that is enough for clinicians to analyze the optimal slice for Alzheimer's disease MRI diagnosis.

We also measured the area of the coronal slice of the skull at the same level as the area of the brain (at CA) to do experimental area normalization to the brain/skull ratio. We found a significantly lower brain area/skull area ratio in the AD group compared to the control group. Furthermore, another experimental normalization of hippocampal areas (left and right) to the brain area/skull area ratio showed similar results as the hippocampus to area of brain in CA normalization—significantly lower in the AD group compared to the control one. The same results showed the brain volume/skull volume ratio and normalization of the hippocampal volumes to brain volume/skull volume ratio (unpublished results). We did not include the above-mentioned data in the article because of similarity of results they revealed.

The limitations of our study include the normalization style we used. We normalized left and right hippocampal areas and volumes to the total brain area at the level of CA and total intracranial volume. TIV normalization does not make a difference between left and right hemispheres so that it compares right and left hippocampal volumes to the volume of the whole brain. More precise and valuable for the statistics would be comparison of hippocampal areas and volumes (left and right) to the area and volume of the corresponding left and right hemispheres, but this is not widely accepted.

## 5. Conclusion

We present a simplified protocol for hippocampal atrophy evaluation on MRI in Alzheimer disease based on single optimal coronal slice analysis. In order to prove it, we measured the absolute area of the hippocampus at the single optimal slice and compared it with the normalized area of the same slice. We found no difference between the hippocampal absolute and normalized area in control and AD patients. Similarly, we did not find a difference between absolute and normalized hippocampal volumes. We found that estimation of hippocampal shrinkage in Alzheimer disease on MRI could be reliably done without normalization.

## Figures and Tables

**Table 1:** Characteristics of participants and group comparisons.

	AD group	Control group	*p* values
Numbers of participants	40	40	
Age at scan (years)	70.3 ± 6.8	67.8 ± 4.7	n.s.
Education (years)	13 ± 3	14 ± 3	n.s.
Male/female sex	13/27	15/25	n.s.
MMSE score (0-30 points)	21 ± 1	29 ± 4	<0.001

Data are expressed as the mean ± standard deviation. MMSE: Mini-Mental State Examination; n.s.: not significant.

**Table 2:** Absolute and normalized hippocampal areas and volumes in Alzheimer disease patients and controls.

Hippocampal measures	AD patients	Controls	*p* values	Cohen's *d*	AUC (SE)
Absolute measures (mm^2^, mm^3^)					
Area of the optimal slice on the left	118.1 ± 38.8	181.1 ± 25.7	<0.01	1.91	0.91 (0.0331)
Area of the optimal slice on the right	112.1 ± 40.2	169.6 ± 29	<0.01	1.64	0.87 (0.039)
Volume on the left (FreeSurfer)	2651 ± 746	3677 ± 948	<0.01	1.2	0.86 (0.0486)
Volume on the right (FreeSurfer)	2777 ± 930	3761 ± 760	<0.01	1.16	0.83 (0.0506)
Normalized measures (%)					
Area of the optimal slice on the left to brain area at anterior commissure	45.5 ± 11.8	63.6 ± 8.7	0.01	1.75	0.89 (0.034)
Area of the optimal slice on the right to brain area at anterior commissure	43.2 ± 13.3	59.5 ± 8.5	<0.01	1.46	0.85 (0.0416)
Volume on the left to TIV (FreeSurfer)	0.29 ± 0.09	0.35 ± 0.08	0.01	0.7	0.73 (0.061)
Volume on the right to TIV (FreeSurfer)	0.31 ± 0.12	0.42 ± 0.14	<0.01	0.84	0.78 (0.0551)

Absolute hippocampal areas in the optimal slice on the left and right are in mm^2^. Absolute hippocampal volumes on the left and right are in mm^3^. Normalized hippocampal areas of the optimal slice to the brain area at anterior commissure on the left and right and normalized hippocampal volumes to total intracranial volume (TIV) on the left and right are in percentages. All values are expressed as means ± standard deviations; SE means standard error of AUC (ROC).

**Table 3:** No differences were found between absolute and normalized measures using comparisons of areas under the receiver operating characteristic curves.

Absolute vs. normalized hippocampal measures	*p* value
Area on the left	0.7
Area on the right	1.0
Volume on the left	0.9
Volume on the right	0.5

## Data Availability

All analyzed data are available upon request in the Institute of Anatomy, Third Faculty of Medicine, Prague, Czech Republic.

## Abbreviations

AD: Alzheimer's disease
AUC: Area under the curve (receiver operating characteristic)
CA: Anterior commissure
FS: FreeSurfer
MCI: Mild cognitive impairment
MRI: Magnetic resonance imaging
MMSE: Mini-Mental State Examination
rWTH: Radial width of the temporal horn
ROC: Receiver operating characteristic
TIV: Total intracranial volume.
